# Supplementary material for: Causal Relationship between Aging and Anorexia Nervosa: A White-Matter-Microstructure-Mediated Mendelian Randomization Analysis
Source: Biomedicines. 2024 Aug 16;12(8):1874. doi: 10.3390/biomedicines12081874 (PMC11351342; doi:10.3390/biomedicines12081874)
Supplement: Supplementary file 1 [file biomedicines-12-01874-s001.zip › Supplementary Figure/Figure S8/Leaveoneout plot of MR analysis for TL to ubm-b-1485.pdf]

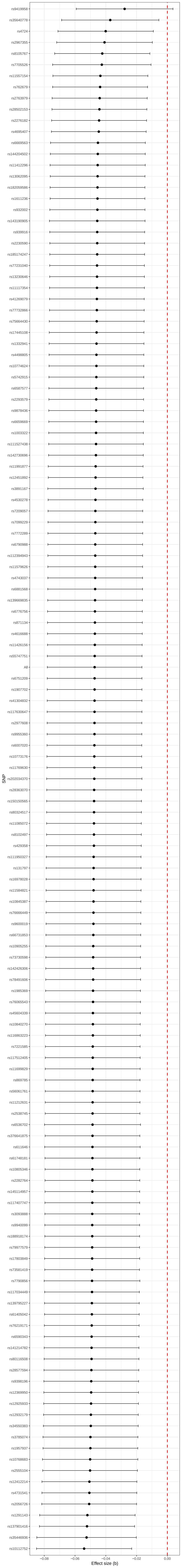

rs9419958
rs35640778
rs4724
rs2967355
rs8105767
rs7705526
rs11557154
rs762679
rs2763979
rs28502153
rs2276182
rs4695407
rs6669563
rs144204502
rs11412296
rs13062095
rs182059586
rs1611236
rs932002
rs143190905
rs939916
rs2230590
rs185174247
rs77231040
rs13230646
rs11117354
rs41269079
rs77732866
rs75664430
rs17445108
rs1332941
rs4498805
rs10774624
rs5742915
rs6587577
rs2293579
rs9878436
rs6659669
rs1003322
rs111527438
rs142730696
rs11991877
rs12451892
rs3891167
rs4530278
rs7209057
rs7099229
rs7772289
rs6790988
rs112394943
rs11579626
rs4743037
rs6881568
rs139669835
rs6776756
rs871134
rs4616688
rs11426156
rs55747751
All
rs6751209
rs1907702
rs41304832
rs117630647
rs2977608
rs9955360
rs6007020
rs10773176
rs11769630
rs202034370
rs28363070
rs150150565
rs80324517
rs11085072
rs8102497
rs429358
rs111950327
rs131797
rs16978028
rs11584821
rs10845387
rs76666449
rs9600019
rs66731853
rs10905255
rs73730598
rs142426306
rs78491606
rs1985369
rs76065543
rs45604339
rs10840270
rs116863223
rs7221585
rs117512405
rs11699829
rs869785
rs56061761
rs11212631
rs2538745
rs6536702
rs376641875
rs611646
rs61748181
rs10805346
rs2282764
rs145114957
rs117407747
rs3093888
rs9940099
rs188918174
rs79977579
rs17803849
rs73581419
rs7790856
rs117034449
rs139795227
rs61405042
rs76219171
rs6590343
rs141214782
rs80116508
rs28577594
rs9398196
rs12369950
rs12925933
rs12932179
rs34550383
rs3785074
rs1957937
rs10768683
rs2555104
rs12412214
rs4731541
rs2056726
rs1291143
rs137901416
rs35446936
rs10112752
SNP
−0.08
−0.06
−0.04
−0.02
0.00
Effect size (b)
